# Performance exploration of multi-gene panels of alopecia areata susceptibility and drug-binding targets

**DOI:** 10.3389/fphys.2025.1489907

**Published:** 2025-03-27

**Authors:** Hongye Liu, Yang Li, Ling Ren, Xiaofeng Yang, Shuo Zhang, Hongmei Bi, Hongxia He, Jingyu Ren, Xiaoqing Lang, Shuping Guo

**Affiliations:** ^1^ Department of Dermatology, The First Hospital of Shanxi Medical University, Taiyuan, Shanxi, China; ^2^ Department of Dermatology, Qingdao Municipal Hospital, Qingdao, Shandong, China

**Keywords:** immune cell infiltration, multi-gene panel, alopecia areata, machine learning, module eigen genes, molecular docking, molecular dynamics simulation

## Abstract

**Objective:**

This study aims to identify potential target genes and therapeutic drugs for alopecia areata (AA).

**Methods:**

Utilizing training and testing data, we evaluated multi-gene panels derived from commonly upregulated genes in publicly available AA patient datasets. The functions of these genes in biological processes were analyzed to identify special multi-gene panels that may play crucial roles in AA. Differences in immune cell infiltration between AA patients and healthy controls were assessed using gene set variation analysis (GSVA) and the Wald test. Signature genes were further validated in specific subsets using single-cell RNA sequence data. Finally, molecular docking and molecular dynamics simulation were conducted to evaluate interactions between protein structures encoded by signature genes and the potential new drug candidates.

**Results:**

When the cut-off value of log_2_FoldChage was greater than 1.0, 51 common upregulated genes were identified in the datasets GSE68801 and GSE45512, and the enrichment analysis of biological process indicated the significant involvement of immune cells in AA. The predictive performance of multi-gene panels demonstrated excellent accuracy in pathways related to “regulation of T cell-mediated cytotoxicity” and “cell killing.” GSVA and the Wald test demonstrated that the infiltration of T cells and NK cells in AA patients was significantly higher than in healthy controls. Based on single-cell immune cell subsets, we found that within the macrophage migration inhibitory factor signaling pathway, the interactions between NK T cells, CD8 T cells, and melanocytes were observed exclusively in AA patients but not in healthy controls. This indicates that NK T and CD8 T cells may play an important role in the attack on hair follicles via melanocytes. Additionally, we selected several important biomarkers for molecular docking with interacting chemicals, evaluated the stability of drug–protein binding patterns through molecular dynamics simulation, and identified several potential targeted therapeutic agents.

**Conclusion:**

In this study, we screened several key genes associated with immune cells and potential drug-like chemicals that could serve as targeted therapies for AA.

## 1 Introduction

Alopecia areata (AA) is a common autoimmune, non-cicatricial form of alopecia and is the second most prevalent type of alopecia in clinic (McMichael and Roberson, 2023; Anzai et al., 2019). Current evidence suggests that AA is primarily a T cell-mediated disease (Anzai et al., 2019; Xing et al., 2014); notably, *in vivo* experiments demonstrated that CD8^+^ T-cell depletion can reverse established AA, whereas CD4^+^ T-cell depletion does not exhibit the same effect (Xing et al., 2014; Lee et al., 2023a). However, the extent of hair loss and the patient’s age are the most significant factors affecting treatment outcomes. Currently, there is no effective treatment for patients with AA, and relapse is quite common following the withdrawal of medication (Xie et al., 2022; Strazzulla et al., 2018).

Corticosteroids, such as triamcinolone acetonide, are commonly used to treat patients with limited AA. In a study involving 219 Asian patients with AA, more than 50% experienced scalp improvement after 12 weeks of treatment (Tan et al., 2002). Another monotherapy with 3%–5% minoxidil was insufficient to promote complete hair regrowth, but it stimulated hair growth in AA patients (Price, 1987). Methotrexate may be an effective treatment for patients with AA who have not responded to standard therapies, and it has shown success in both adults (Chartaux and Joly, 2010) and children (Royer et al., 2011). In a recent double-blind randomized clinical trial (NCT02037191) (Joly et al., 2023), 45 out of 89 AA patients received methotrexate therapy, and hair regrowth was partially restored in patients treated with methotrexate alone, while complete regrowth was achieved in up to 31% of patients who received a low-dose combination with prednisone. Furthermore, Janus kinase (JAK) inhibitors, such as tofacitinib, ruxolitinib, and baricitinib, have demonstrated efficacy in treating AA patients. However, the durability of the response to these medications is erratic, and most patients experience hair loss again after discontinuation (Mackay-Wiggan et al., 2016a). Therefore, it is essential to accelerate the development of new therapeutic targets and drugs to help AA patients regain full hair and restore their confidence.

In this study, we first screened significant genes associated with immune cell function and developed training and testing models to validate their role in identifying AA samples. Subsequently, we simulated the drug-binding scores for pockets in the crystal structure and evaluated the affinity scores of target gene-interacting chemicals to identify potential drugs for AA patients.

## 2 Methods and materials

### 2.1 Collection of AA data and candidate genes

AA-associated bulk-RNA sequence datasets [GSE68801 (Jabbari et al., 2016), GSE45512 (Xing et al., 2014), and GSE80342 (Mackay-Wiggan et al., 2016b)] and single-cell RNA sequence dataset (GSE233906) were manually downloaded from Gene Expression Omnibus (GEO). Detailed characteristics of the patients and samples used in this study are provided in Supplementary Table S1. The differentially expressed genes (DEGs) between AA patients and healthy controls in the GSE68801 and GSE45512 datasets were identified using Wald significance tests from the DESeq2 package (version 1.42.0) in R language, based on a negative binomial distribution (Love et al., 2014). Potential candidate genes were screened from the shared upregulated DEGs with log_2_FoldChange greater than 1.00 and *P* value less than 0.05.

### 2.2 Evaluation of multi-gene panel performance

First, an enrichment analysis of biological processes was performed for shared upregulated DEGs to identify relevant multi-gene panels, each containing a maximum of four genes. Then, a generalized linear model was applied to the training dataset GSE68801, and the performance of the corresponding gene panel was tested in other datasets GSE45512 and GSE80342. Finally, to systematically train and test the performance of multi-gene panels in predicting AA patients, 97 machine learning models were deployed to find optimal multi-gene panels. Detailed methods and models are provided in the reproducible code.

### 2.3 Comparison of immune cell infiltration

To explore the different roles of immune cells played in AA patients, the GSVA (Hänzelmann et al., 2013) was conducted to compare immune cell infiltration in individual samples based on the CIBERSORT and TCIA signature genes identified by Newman et al. (2015) and Charoentong et al. (2017), which include 22 and 28 gene sets representing different immune cell types and/or functions, respectively. Then, the Wald test of individual scores was applied between AA patients and healthy controls, determining statistical significance and fold-change differences.

### 2.4 Correlation analysis between eigen genes and immune cell infiltration

The weighted gene co-expression network analysis (WGCNA) of the top 10,000 upregulated or downregulated DEGs was conducted to find potential module eigen genes (Langfelder and Horvath, 2008). In the training data of this study, a soft thresholding power value of 10 was selected for fitting the unsigned scale-free topology network. The adaptive pruning algorithm based on hierarchical clustering with a minimum size of 100 genes was used to calculate the topological overlap matrix and corresponding dissimilarity. Then, the consensus measure in the gene expression network, defined by the correlation of the eigen genes, was used to merge modules with a correlation greater than 0.80 into a new gene module. Finally, the Pearson correlation matrix of module eigen genes and immune cell infiltration was generated and visualized on a heatmap.

### 2.5 Identification of signature genes

To verify the differences in signature genes among subtypes of immune cells, we compared the target gene expression in a publicly available single-cell RNA sequence dataset (GSE233906), including six AA patients and two healthy controls. The initial cell clusters were recognized using Seurat V5 (Hao et al., 2024), and cell types were annotated using the reference Human Primary Cell Atlas Data in the R package SingleR (Version 2.4.1) (Aran et al., 2019). The Wilcox test was then used to compare the differences in signature genes in subset cells between AA patients and healthy controls.

### 2.6 Evaluation of drug-binding performance

To evaluate the potential targeted therapeutic properties of these signature genes, crystal structures of target genes were acquired from the Worldwide Protein Data Bank (PDBe-KB, 2022). The identification of drug-binding pockets followed a Voronoi mosaic-based protein pocket detection algorithm (Le Guilloux et al., 2009). Only pockets with drug score greater than 0.1 were used for molecular docking evaluation. The docking program smina, based on the AutoDock Vina tool, was used to predict receptor–ligand binding affinity (Koes et al., 2013). The potential binding chemicals were queried from the Comparative Toxicogenomics Database (CTDdb) (Davis et al., 2023).

### 2.7 Molecular dynamics simulation

To further understand the stability and flexibility of drug binding to target chemicals, we performed molecular dynamics simulations using GROMACS (2024) (Pronk et al., 2013). Coordinate and topological files were compiled in GROMACS using the AMBER94 force field (Sorin and Pande, 2005) and the recommended water model TIP3P (transferable intermolecular potential with 3 points). Physiological conditions were simulated by adding 0.15 M of salt (NaCl) in a 1 cubic nanometer solvent box. During all simulations, each protein–ligand complex was simulated for 300 ns at 300 K and 1 atm pressure. Free energy landscape (FEL), root mean square deviation (RMSD), root mean square fluctuation (RMSF), and solvent-accessible surface area (SASA) were used to evaluate the thermal and pressure stability and flexibility of the binding chemicals.

### 2.8 Statistics and reproducibility

All statistical analyses were conducted on platforms R-4.3.2 and Python-3.9.10. The functional enrichment analysis for biological processes of gene sets was calculated using the R package clusterProfiler (Version 4.10.1) (Wu et al., 2021). WGCNA (Version 1.72-5) (Langfelder and Horvath, 2008) was used to calculate the correlation between eigen genes of DEGs and scores of immune cell infiltration. The crystal structures of drug-binding pockets and molecular docking were visualized using the Python packages py3Dmol (Version 2.0.4) and pymol (Version 2.5.7).

## 3 Results

### 3.1 Common DEGs revealed the important role of immune cells in AA

**FIGURE 1:**
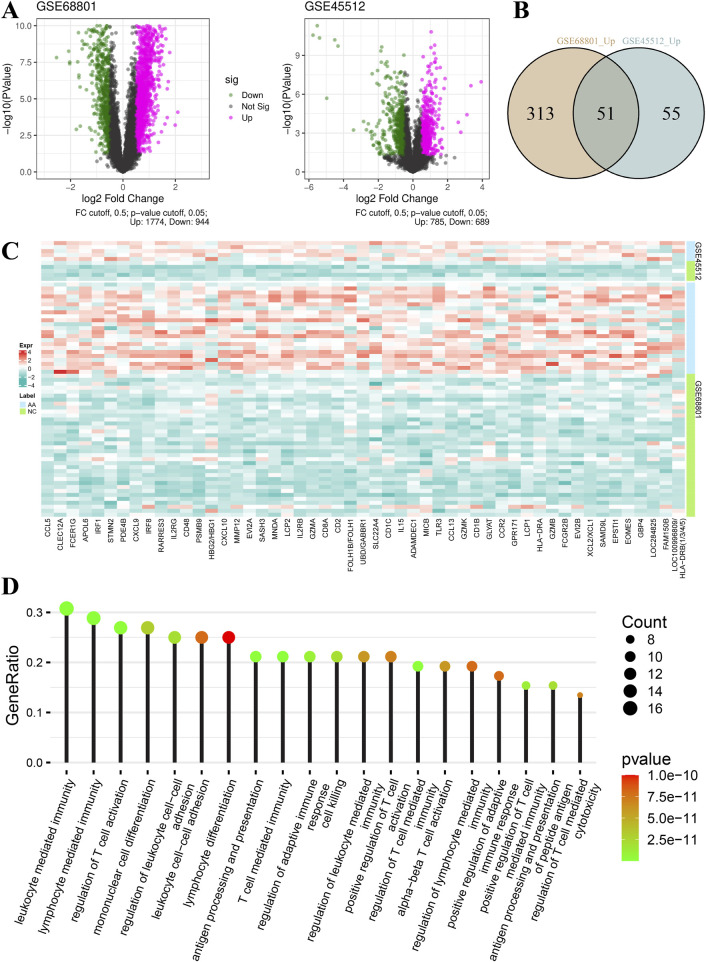
Gene expression and biological function of signature genes. **(A)** Volcano plots of differentially expressed genes of datasets GSE68801 and GSE45512. **(B)** Venn plot of common upregulated genes of datasets GSE68801 and GSE45512. **(C)** Relative expression heatmap of 51 common genes (AA, alopecia areata; NC, normal control). **(D)** Top 20 terms in enrichment results of biological function for the 51 common genes. The dot size and color represent the gene count and P value of each term, respectively.

As shown in Figure 1A, at a cut-off value of 0.5 for log_2_FoldChange, 1,774 genes were upregulated and 944 genes were downregulated in the GSE68801 dataset, 785 genes were upregulated and 689 genes were downregulated in the GSE45512 dataset. When the cut-off value of log_2_FoldChange was 1.00, 364 and 106 genes were upregulated in GSE68801 and GSE45512, respectively, of which 51 were common genes (Figure 1B; Supplementary Table S2). Figure 1C shows the relative expression values of these common genes in the individual samples. Interestingly, the enrichment of biological process for these 51 genes was mainly associated with immune cell regulation, differentiation, and activation (Figure 1D; Supplementary Table S3). For example, processes such as T-cell activation regulation and lymphocyte differentiation were significantly enriched.

### 3.2 Predictive performance of multi-gene panels

**FIGURE 2:**
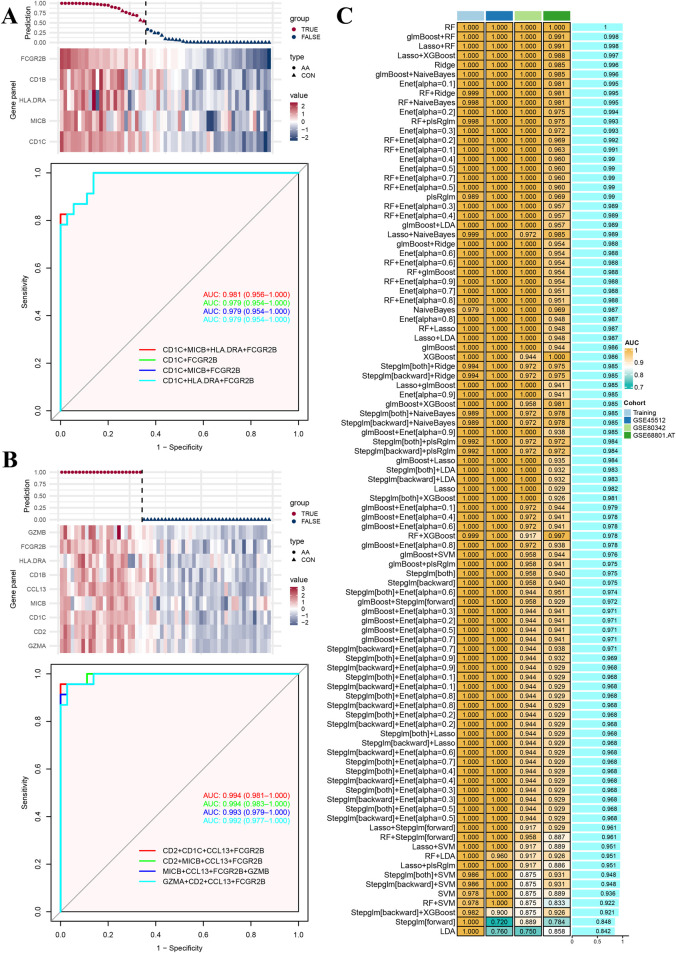
Model training and testing results. **(A)** Prediction model and relative expression heatmap for genes in the enriched term “regulation of T cell-mediated cytotoxicity.” Performance of top four multi-gene panel receiver operative curves. **(B)** Prediction model and relative expression heatmap for genes in the enriched term “cell killing.” Performance of top four multi-gene panel receiver operative curves. **(C)** Performance of training and testing cohorts for 97 machine learning models.

To assess the predictive performance of multi-gene panels, candidate genes associated with “regulation of T cell-mediated cytotoxicity” and “cell killing” were chose to fit generalized linear models on individuals from the GSE68801 dataset. As shown in Figure 2A, the multi-gene panel consisted of *CD1C*, *MICB*, *CD1B*, *FCGR2B*, and *HLA*-*DRA* genes, and the predicted results included four (4/23) false-positive individuals and three (3/36) false-negative individuals. The AUC values of the top four combination of multi-gene panels were all greater than 0.95. In another multi-gene panel consisting of *GZMA*, *CD2*, *CD1C*, *MIC*, *CCL13*, *CD1B*, *HL*A-*DRA*, *GZMB*, and *FCGR2B* genes (Figure 2B), none of the individuals were predicted to be false-positive or false-negative. The AUC values of the top four combination of multi-gene panels were also greater than 0.95. Furthermore, the same multi-gene panels were tested in other datasets GSE45512 and GSE80342 (Supplementary Figure S1A, B), and the predictive performance was even better than that of the current training dataset.

In addition, to avoid over-fitting within the multi-gene panels, a series of machine learning algorithms, including 97 different combinations, were used for training and testing in different datasets to screen the optimal fitting models. As shown in Figure 2C, based on the aforementioned 51 common genes, most of the fitted models demonstrated strong predictive performance in both the training or testing cohorts, although some models exhibited false-negative or false-positive scores in certain samples (Supplementary Figure S2).

### 3.3 Infiltration of immune cells in AA

**FIGURE 3:**
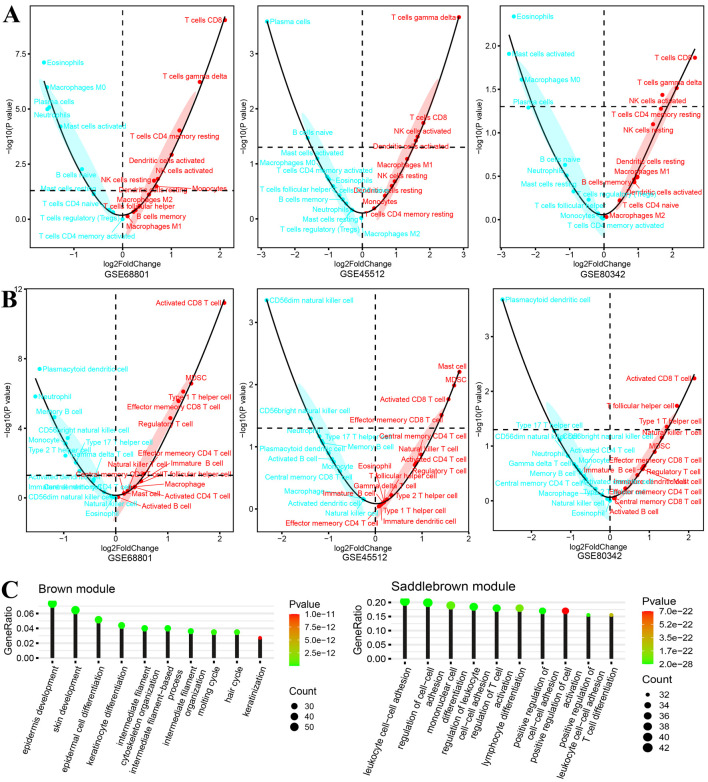
Differences in immune cell infiltration and biological function of gene modules of interest. **(A, B)** Significance tests and fold changes for immune cell infiltration [**(A)** CIBERSORT and **(B)** TCIA] scores of gene expression sets from the scalp of AA patients and healthy controls in datasets GSE68801, GSE45512, and GSE80342. **(C)** Biological functions of two gene modules (negatively correlated brown module and positively correlated saddle brown module), which focused on skin cell development and immune cell activation, respectively.

We further performed GSVA on all included samples to evaluate the degree of infiltration of different types of immune cells. As shown in Figure 3A, in the three cohorts of CIBERSORT immune cell infiltration results, the infiltration scores of “T cell gamma delta,” “T cell CD8,” “NK cells activated,” and “NK cells resting” in AA individuals were significantly increased. On the other hand, the infiltration scores of “eosinophils”, “plasma cells,” and “mast cells activated” were significantly lower than those in healthy controls. As shown in Figure 3B, the infiltration score changes between AA patients and healthy controls in TCIA immune cell subtypes suggest the important role of CD8 T cells, such as “activated CD8 T cell” and “effector memory CD8 T cell.” Consistent with a previous study, the depletion of CD8 T cells reversed the AA disease in an *in vivo* experiment in mice (Lee et al., 2023a). All immune cell infiltration scores were used as phenotypic traits to calculate associations with module eigen genes.

### 3.4 Correlation of eigen genes and immune cell infiltration

**FIGURE 4:**
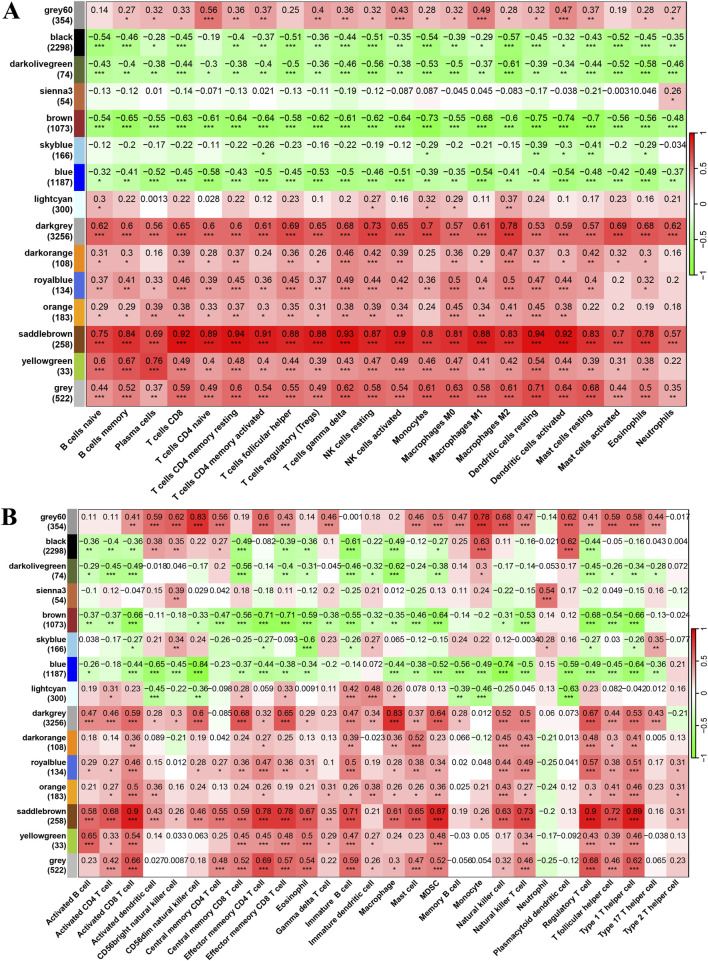
Pearson correlation results of module eigen genes and immune cell infiltration scores. **(A, B)** Heatmap of Pearson correlation results of module eigen genes and immune cell infiltration scores. [**(A)** CIBERSORT and **(B)** TCIA; *P < 0.05, **P < 0.01, ***P < 0.001].

In the final results of merged WGCNA, 15 gene modules were found within the selected DEGs in the training cohort. Interestingly, the biological process enrichment analysis of each gene module revealed that the brown module was significantly enriched in “epidermis development,” “epidermal cell differentiation,” “keratinocyte differentiation,” and “hair cycle” and that the saddle brown module was significantly enriched in “leukocyte cell–cell adhesion,” “mononuclear cell differentiation,” “regulation of T-cell activation,” and “T-cell differentiation” (Figure 3C). Moreover, the significant correlation results between module eigen genes and CIBERSORT immune cell infiltration indicate that they play important roles in these biological processes. As shown in Figure 4A, a few gene modules were significantly associated with all immune cell infiltration scores, such as positively correlated modules gray, dark-gray, and saddle brown, and negatively correlated modules blue, brown, and dark olive-green. In addition, the correlation results of module eigen genes are generally consistent with the TCIA immune cell atlas compared with similar cell subtypes (Figure 4B).

### 3.5 Immune cell subsets and signature genes

**FIGURE 5:**
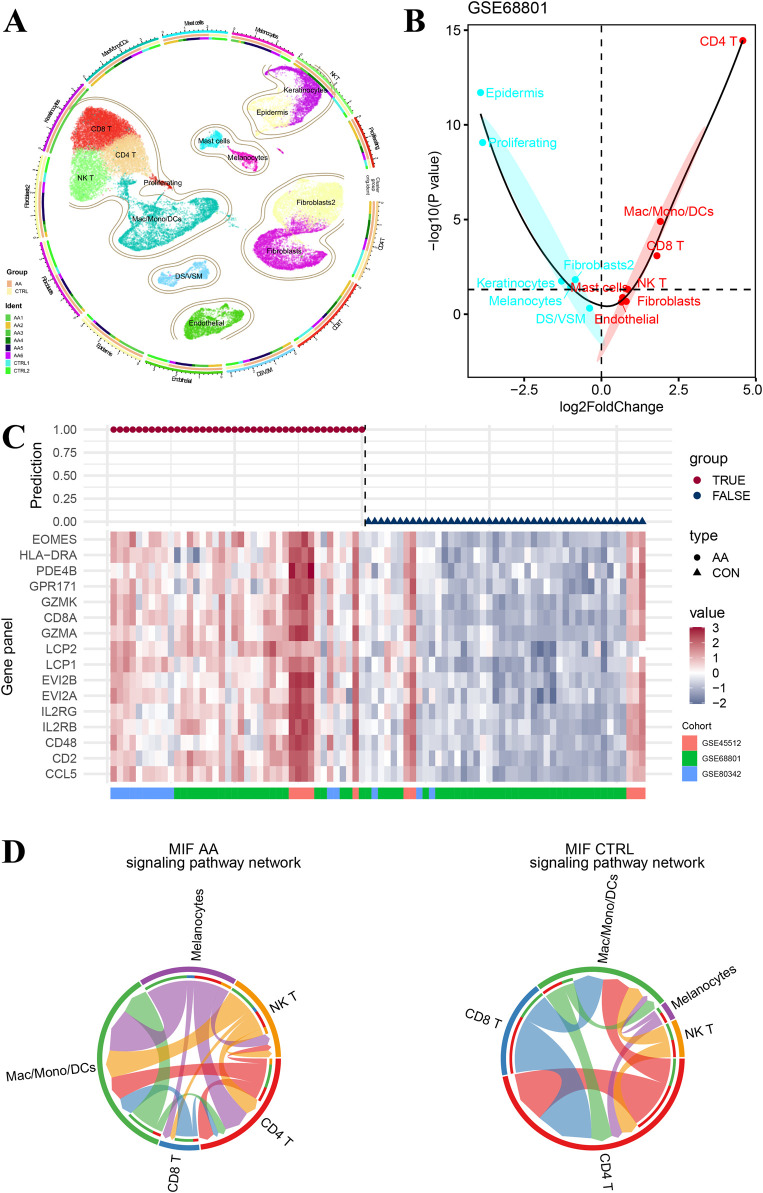
Immune cell interactions in single cell RNA-seq cell subtypes. **(A)** Cell type atlas of six alopecia areata patients and two healthy controls in the single-cell RNA sequence dataset GSE233906. **(B)** Difference in cell type infiltration on the basis of top 50 biomarkers in each cell cluster. **(C)** Predictive performance of gene-panel with 16 genes in pooled datasets GSE68801, GSE45512, and GSE80342. **(D)** Comparative network of cell–cell chat (including CD4 T, CD8 T, NK T, mac/mono/DCs, and melanocyte) signaling pathways for macrophage migration inhibitory factors.

Figure 5A shows the single-cell UMAP plot of the different cell subsets and the relative proportions of cell subtypes in six AA patients and two healthy controls. Then, we screened 16 genes that were highly expressed in immune cell subtypes such as CD4 T, CD8 T, and NK T (Supplementary Figure S3A). The Wilcox test of signature genes was conducted between AA patients and healthy controls in different cell subtypes (Supplementary Figure S3B), and GSVA scores of samples based on the top 50 biomarkers in each cell cluster were compared (Figure 5B; Supplementary Table S4). Consistent with the aforementioned result, the immune cell infiltration scores of subtypes CD4 T, CD8 T and NK T were significantly increased in AA patients. In addition, a generalized linear model of the 16 genes was fitted in cohorts GSE68801, GSE45512 and GSE80342, and there were no false-positive or false-negative predictions in the results (Figure 5C).

There is evidence that melanocyte peptide epitopes can function as autoantigens to induce NK cells and T lymphocytes accumulated around hair follicles in the lesional skin of AA patients but rarely in the normal skin due to the immune privilege (Ito et al., 2008; Gilhar et al., 2001). Therefore, we constructed a network of cell–cell chat (including CD4 T, CD8 T, NK T, Mac/Mono/DCs, and melanocyte) signaling pathways for macrophage migration inhibitory factors (Figure 5D). As a result, signaling from NK T and CD8 T cells to melanocytes was observed in AA patients but not in healthy controls, suggesting that NK T and CD8 T may play an important role in attacking hair follicles via melanocytes.

### 3.6 Drug-binding properties of proteins encoded by signature genes

**FIGURE 6:**
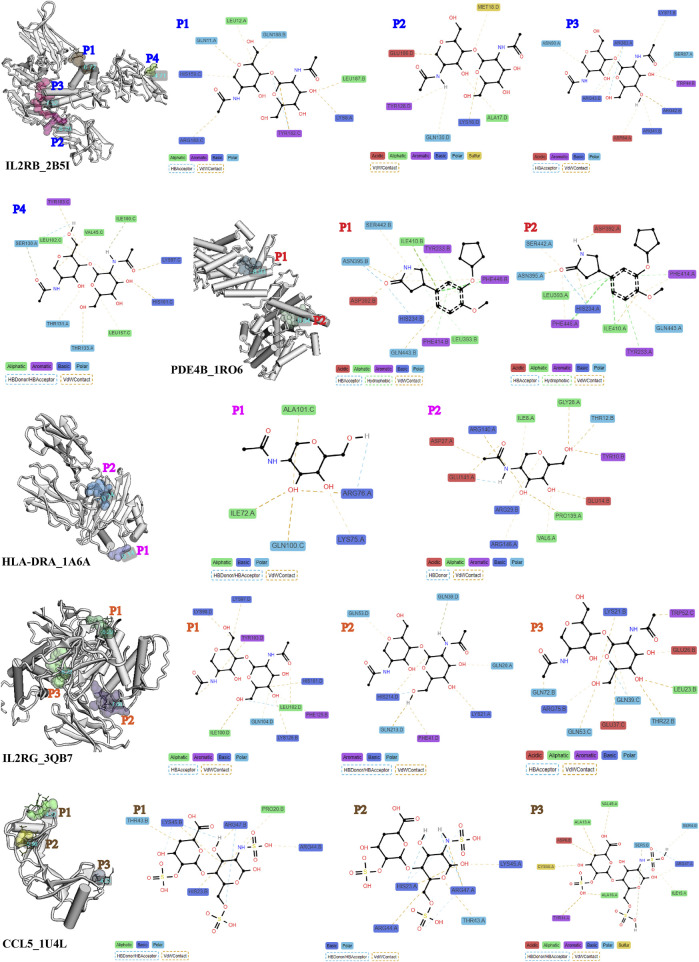
Details of ligand–residue interactions in drug-binding pockets.

**FIGURE 7:**
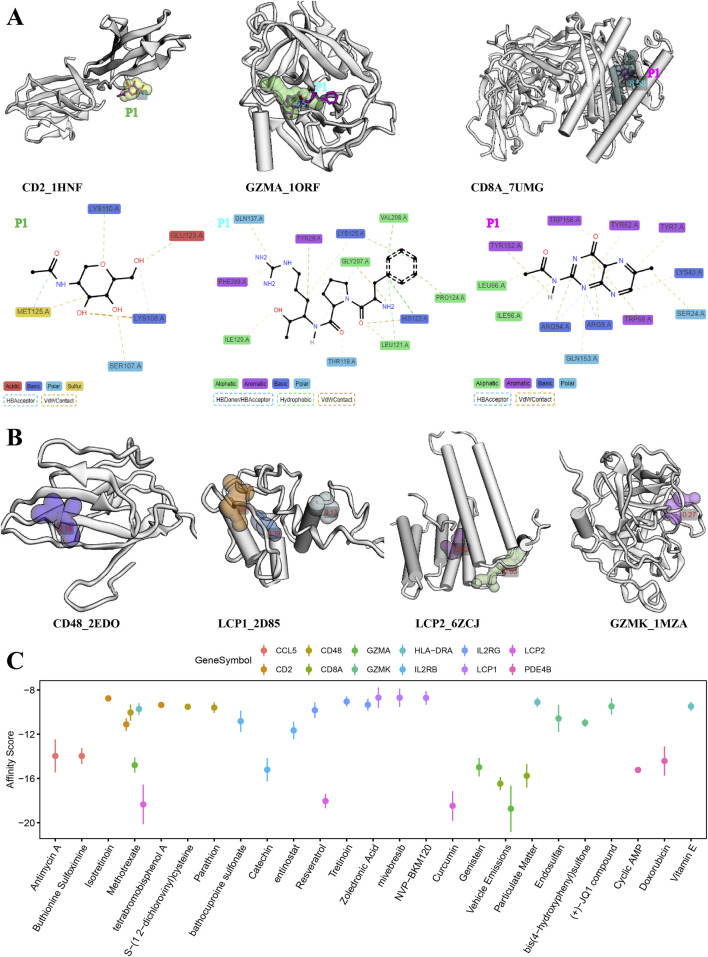
Evaluation of molecular docking and drug-binding pocket. **(A)** Details of ligand–residue interactions in drug-binding pockets. **(B)** Crystal structure of the optimal molecular docking position in each available pocket. **(C)** Top three chemicals with the highest affinity score for protein molecular encode by each signature gene.

The evaluation of target therapeutic properties was based on the drug-binding score of the pocket and molecular docking affinity. As shown in Figures 6, 7A, the molecular structure of eight proteins, namely, CCL5, CD2, IL2RB, IL2RG, PDE4B, GZMA, CD8A, and HLA-DRA, and the details of ligand–residue interactions in the drug-binding pocket only presented the optimal molecular docking pose in each binding pocket. The four other genes, namely, *CD48*, *LCP1*, *LCP2*, and *GZMK*, in Figure 7B, only presented the available pockets in their protein structures with drug-binding score greater than 0.1 (Supplementary Table S5). The remaining four genes, namely, *EOMES*, *GPR171*, *EVI2A*, and *EVI2B*, do not have corresponding 3D crystal structures of proteins in the database.

In addition, we searched the CTDdb to look for therapeutic chemicals associated with target genes (Supplementary Table S6). Molecular docking was performed between available molecular binding pockets and interacting chemicals, as shown in Figure 7C, targeting the top three chemicals with the highest affinity score for each gene. In general, the higher the energy score, the stronger the binding ability between the two molecules and the greater the complexity of the interaction. For example, the chemicals methotrexate, resveratrol, and curcumin are the most optimal target-binding drugs for the *LCP2* gene. Details of the molecular docking results are provided in Supplementary Table S7.

### 3.7 Stability of bonded composites

**FIGURE 8:**
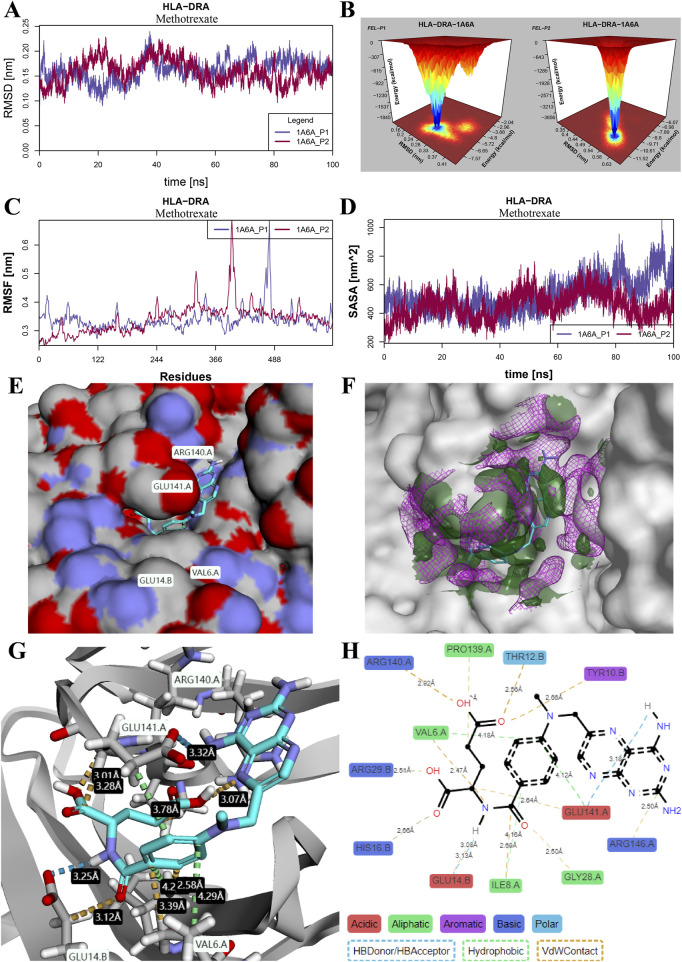
Stability evaluation of molecular dynamics simulation for the binding mode of methotrexate and HLA-DRA protein. **(A)** RMSD changes in the two binding pocket modes. **(B)** Three-dimensional heatmap of the cumulative distribution of free energy landscapes (FELs) over RMSD variations in a 300-nanosecond molecular dynamics simulation. **(C)** RMSF of all amino acid residues in each binding pocket mode. **(D)** SASA changes in the two binding pocket modes. **(E)** Binding conformation of methotrexate and HLA-DRA protein complex. **(F)** Free energy diagram of solvent stability and contribution of solvation to binding free energy. The dark-green area and violet grid represent free energies less than −0.2 kcal/mol/Å^3^ and greater than 0.2 kcal/mol/Å^3^, respectively, at a distance of 5Å from the ligand. **(G, H)** Interactions of methotrexate with binding residues in the crystal structure (G) and schematic diagram (H) of the HLA-DRA protein.

An example of molecular dynamics simulation of the binding mode of methotrexate to the HLA-DRA protein is shown in Figure 8. The variation in RMSD in stable ranges reflects the stability of the simulation system (Figure 8A). The cumulative distribution of FEL over RMSD in 300 nanoseconds simulation time is visualized using 100 grid matrices (Figure 8B), where the aggregated peaks correspond to the stability pattern of the composite. The overall RMSF of all amino acid residues is shown in Figure 8C, which characterizes the flexibility and motion intensity of protein amino acids during the whole simulation process. Figure 8D shows the changes in SASA of the two drug–protein binding patterns over the simulation time. Detailed simulation results of RMSD, FEL, RMSF, and SASA in other potential drug–protein binding modes are shown in Supplementary Figures S4–7, respectively.

The binding conformation of the methotrexate and HLA-DRA protein complex is shown in Figure 8E. The free energy diagram of solvent stability and contribution of solvation was visualized at a distance of 5 Å from the ligand (Figure 8F), and the large coverage area of the receptor and ligand indicates high solvent stability and accessibility. The total binding energy of the methotrexate molecule with the HLA-DRA protein is −10.49 kcal/mol (Supplementary Table S6). Details of the interaction between amino acid residues and methotrexate atoms were displayed in the crystal structure (Figure 8G) and schematic diagram (Figure 8H) of the HLA-DRA protein, respectively. It was observed that the methotrexate molecule and HLA-DRA protein formed four H-bonds at distances of 3.18 Å, 3.00 Å, 3.12 Å, and 3.13 Å. Three amino acid residues (Val6, Glu141, and Ile8) were involved in the hydrophobic interactions at distances of 4.18 Å, 4.12 Å, and 4.16 Å, respectively.

## 4 Discussion

In this study, we screened 51 upregulated DEGs from the two datasets of AA patients and healthy controls based on log_2_FoldChange greater than 1.0. The predictive performance of the generalized linear binomial model with different multi-gene panels was applied to training and testing datasets. A series of machine learning algorithms, including 97 different combinations, were tested to avoid model over-fitting. The important role of immune cells, especially CD8 T cells and CD4 T cells, in AA patients was confirmed by the infiltration analysis of featured gene sets and the correlation test with the modular eigen genes. In addition, we validated the important role of immune cell subsets in single-cell RNA sequence data and used 16 highly expressed genes in immune cells for further analysis to develop novel target drugs. Finally, evaluations of molecular docking and molecular dynamics simulations were performed between the protein structures encoded by target genes and the interacting chemicals to discover potential new drugs.

Consistent with the known evidence, our study found that CD4+/8+ T cells play an important role in the formation of AA. As described in single-cell RNA sequence data by Lee et al. (2023a), CD8^+^ T cells are the primary disease-driving cell type in AA but not CD4^+^ T cells or NK cells. In a rat model with CD4^+^ T cell deletion, significant hair growth was observed within 23 days of the start of treatment; however, with the re-emergence of CD4^+^ T cells, the newly generated hair was eventually lost (McElwee et al., 1999). Interestingly, in another study of peripheral blood from patients with active AA, the circulating CD4/CD8 T cells and NK cells were significantly reduced compared to healthy controls (Younes et al., 2022). Therefore, whether CD4^+^ T cells act directly on hair follicles or are driven by cytotoxic T lymphocytes via T helper cells requires further investigation. Additionally, there is evidence that cytotoxic CD8+NKG2D+ effector T cells play an important role in the induction of AA in mice (Xing et al., 2014). According to the released cytokines, the CD8+NKG2D+ effector T cells are classified into several subsets, including iNKT1, iNKT2, iNKT10, and iNKT17 (Xu et al., 2023). In an *in vivo* study using a humanized model, Ghraieb et al. (2018) suggested that iNKT10 cells are key disease-protective lymphocytes in AA lesions. However, the function of other subtypes of iNKT in experimental models of AA has not been studied and confirmed.

Aside from immune cells, current evidence suggests that melanocytes are the trigger point for immune system attacks that cause hair follicle destruction (Xie et al., 2022; Gilhar et al., 2001; Przybyla et al., 2021). In a mouse model of lesional scalp grafts on SCID mice by Gilhar et al. (2001), melanocyte-peptide-activated T cells significantly reduced hair regrowth, suggesting that melanocyte-peptide epitopes can function as autoantigens in alopecia areata. In the macrophage migration inhibitor signaling pathway (Figure 5D), cell–cell interactions of melanocytes from other cell types, such as CD8^+^ T and NKT, were only present in AA samples, suggesting that melanocytes may play an important role in AA induction. One possible mechanism is that the autoantigens released during the apoptosis of melanocytes that cannot be repaired in time induce CD8^+^ T-cell attack (Xie et al., 2022).

As for existing treatments for AA patients, both local and systemic, none are effective in the long term, both may have side effects, and relapse often occurs (Zhou et al., 2021). In recent years, with a further understanding of the pathogenesis of AA, some new therapeutic strategies have emerged, such as JAK inhibitors (Mackay-Wiggan et al., 2016a) and some small molecule drugs (Price, 1987; Chartaux and Joly, 2010; Royer et al., 2011; Joly et al., 2023). However, because of the efficacy, side effects, and limited persistence of these drugs, treatment outcomes in patients with AA are often unsatisfactory. Therefore, based on our findings of important target genes and interacting chemicals, we performed molecular docking procedures to search for potential novel therapeutic agents. As a result, molecular methotrexate showed a high affinity for protein structures of LCP2, CD2, CD48, IL2RB, and GZMA; resveratrol showed a high affinity for protein structures of LCP2 and IL2RG; and curcumin showed a high affinity for protein structures of LCP2 (Figure 6C).

Consistent with recent evidence from a randomized controlled trial (NCT02037191) (Joly et al., 2023), the combination of methotrexate and low-dose prednisone resulted in full hair regrowth in up to 31% of patients, with nearly the same effect as the JAK inhibitors. Results from another single-center retrospective case series suggest that methotrexate monotherapy is a viable option for patients with severe AA who do not respond to other standard therapies or have systemic corticosteroid contraindications (Kinoshita-Ise et al., 2021). The same conclusion has been found in systematic reviews and meta-analyses of AA patients receiving methotrexate treatment, and adults appear to be more sensitive to methotrexate treatment than pediatric patients (Phan et al., 2019). However, in another systematic review and meta-analysis of AA patients using JAK inhibitors, there was no difference in response rates between children and adult cases (Phan and Sebaratnam, 2019). In a randomized controlled trial using a mixture of curcumin in the treatment of alopecia areata, curcumin had a 63.33% response rate but was not better than minoxidil (70%) in short-term treatment (12 weeks) (Mao et al., 2022).

Another new highlight in this study is the application of molecular docking and molecular dynamics simulation in the exploration of potential therapeutic drugs to treat AA. For decades, computer-aided screening of novel drugs has played an important role in the treatment of many diseases (Sliwoski et al., 2014; Pinzi and Rastelli, 2019). For example, in the research of a new compound that could reduce the appearance of age spots, Mann et al. (2018) performed molecular docking, screening 50,000 compounds for comparison with known whitening ingredients. In clinical randomized controlled trials, they found that Thiamidol was successful in reducing human skin pigmentation. Ghosh et al. (2018) found that by applying molecular docking to find new treatments for acne, the molecule VCD-004 showed optimal skin penetration and a powerful anti-inflammatory effect by reducing pro-inflammatory cytokines (IL-6), independent of its antibacterial effect (Ghosh et al., 2018). Aside from the molecular docking process, molecular dynamics simulation is an important approach to evaluate the effect of simulated physiological conditions on docked drug–protein complexes (Liu et al., 2018; Parise et al., 2024). In this study, the stability and flexibility evaluation results of RMSD, FEL, RMSF, and SASA advance our understanding of the available drug-like chemicals to target proteins, thereby accelerating the development of new therapeutic agents.

The exact pathological mechanism of AA is not clear, but the widely described theory is a collapse of the immune privilege system, making the functional changes within immune cells in AA pathology highly complex. Therefore, there are certain limitations in discussing the role of immune subsets of cells in AA in this study. For example, the absence or aberrant function of T-reg cells will lead to autoimmune diseases as T-reg cells can suppress the immune responses of other cells and act as master regulators of self-tolerance (Lucca and Dominguez-Villar, 2020). There is evidence that T-reg cells are significantly reduced in patients with AA compared to other dermatoses (Speiser et al., 2019). Uchida et al. (2020) revealed that the number of γδT cells in the scalp of patients with AA was significantly higher than that of healthy controls. IFN-α-producing plasmacytoid dendritic cells initiate the occurrence of AA in mice by inducing cell apoptosis (Ito et al., 2020). In addition, most potential target gene chemicals need to be evaluated and validated in future *in vitro* or *in vivo* experiments before clinical approval.

## 5 Conclusion

As far as we know, AA is a complex autoimmune disease, and further evidence and validation are needed to support a precise theory of its pathogenesis. In any case, this study confirmed the important roles of CD4^+^ T and CD8^+^ T cells in bulk RNA and single-cell RNA sequence datasets; using robust methods to train and test models of key genes, as well as molecular docking and evaluation of protein-chemical evaluations, we screened several important target proteins and drug-like compounds. The findings of this study, in whole or in part, contribute to a better understanding of the characterization of immune cell subsets in AA and provide new insights for future research on targeted therapies.

## Data Availability

The original contributions presented in the study are included in the article/Supplementary Material; further inquiries can be directed to the corresponding authors.
