# Supplementary material for: Performance exploration of multi-gene panels of alopecia areata susceptibility and drug-binding targets
Source: Front Physiol. 2025 Mar 27;16:1489907. doi: 10.3389/fphys.2025.1489907 (PMC11983455; doi:10.3389/fphys.2025.1489907)
Supplement: Supplementary file 1 [file DataSheet1.pdf]

Supplementary Figures Legend

Figure S1. The predictive performance of multi-gene panels in data sets GSE45512 and GSE80342

A. The predictive performance of gene-panel FCGR2B, CD1B, HLA-DRA, MICB and CD1C. **B**. The predictive performance of gene-panel GZMB, FCGR2B, HLA-DRA, CD1B, CCL13, MICB, CD1C, CD2 and GZMA.

Figure S2. Predictive scores of 97 machine learning models and real status of individuals.

Figure S3. Feature plot (A) and Wilcox test (B) result of signature genes

Figure S4. Root mean square deviation changes in molecular dynamic simulations for drug-binding pockets of the proteins encoded by signature genes

Figure S5. Changes in free energy landscape values and root mean square deviation of each drug-binding pocket pattern in a 300-nanosecond Molecular dynamics simulation

Figure S6. Root mean square fluctuation changes in molecular dynamic simulations for drug-binding pockets of the proteins encoded by signature genes

Figure S7. Solvent accessible surface area changes in molecular dynamic simulations for drug-binding pockets of the proteins encoded by signature genes

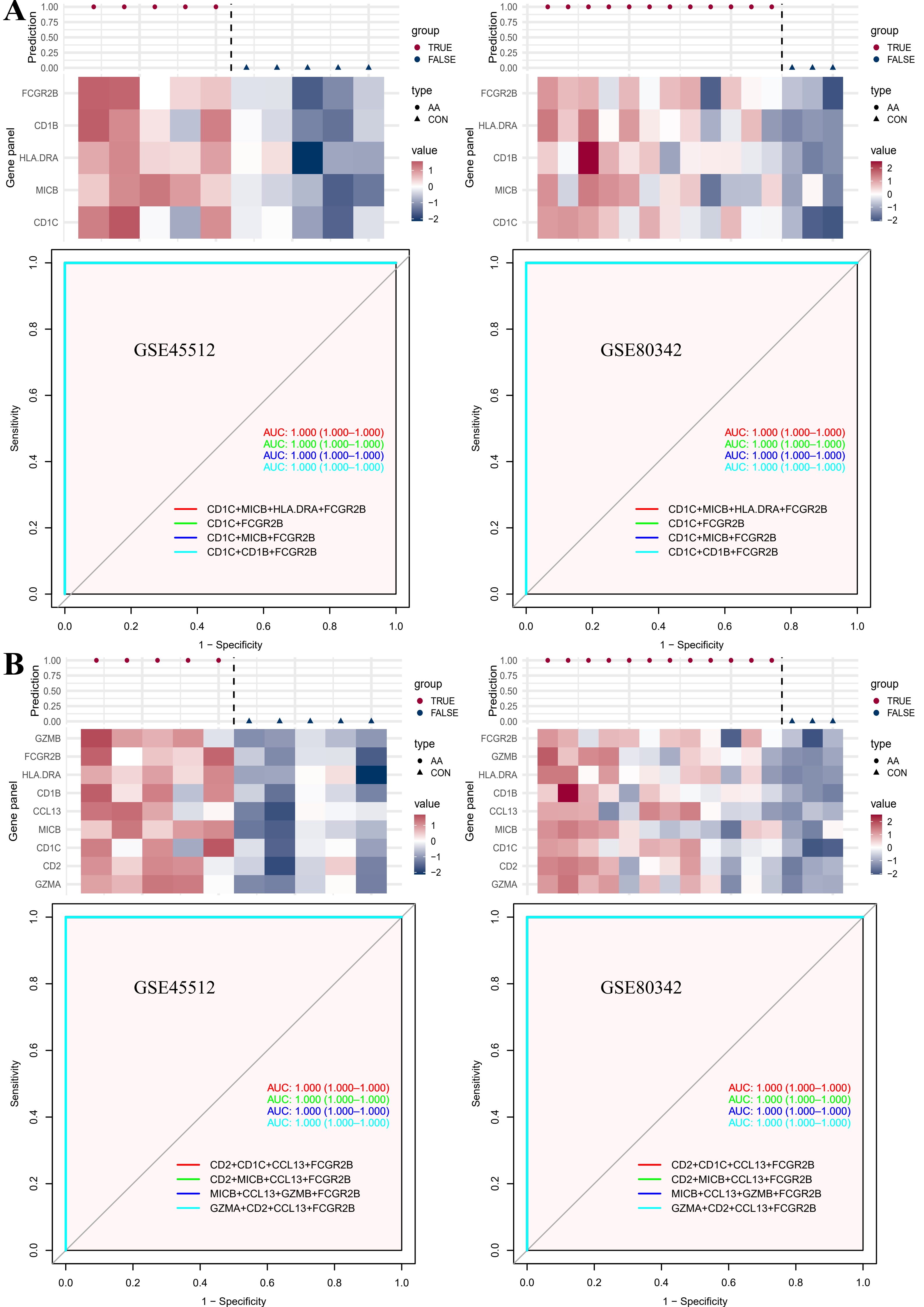
A
B
Prediction
1.00
0.75
0.50
0.25
0.00
group
TRUE
FALSE
type
AA
CON
value
Gene panel
FCGR2B
CD1B
HLA.DRA
MICB
CD1C
GZMB
CCL13
CD2
GZMA
Sensitivity
1 − Specificity
GSE45512
GSE80342
AUC: 1.000 (1.000–1.000)
CD1C+MICB+HLA.DRA+FCGR2B
CD1C+FCGR2B
CD1C+MICB+FCGR2B
CD1C+CD1B+FCGR2B
CD2+CD1C+CCL13+FCGR2B
CD2+MICB+CCL13+FCGR2B
MICB+CCL13+GZMB+FCGR2B
GZMA+CD2+CCL13+FCGR2B

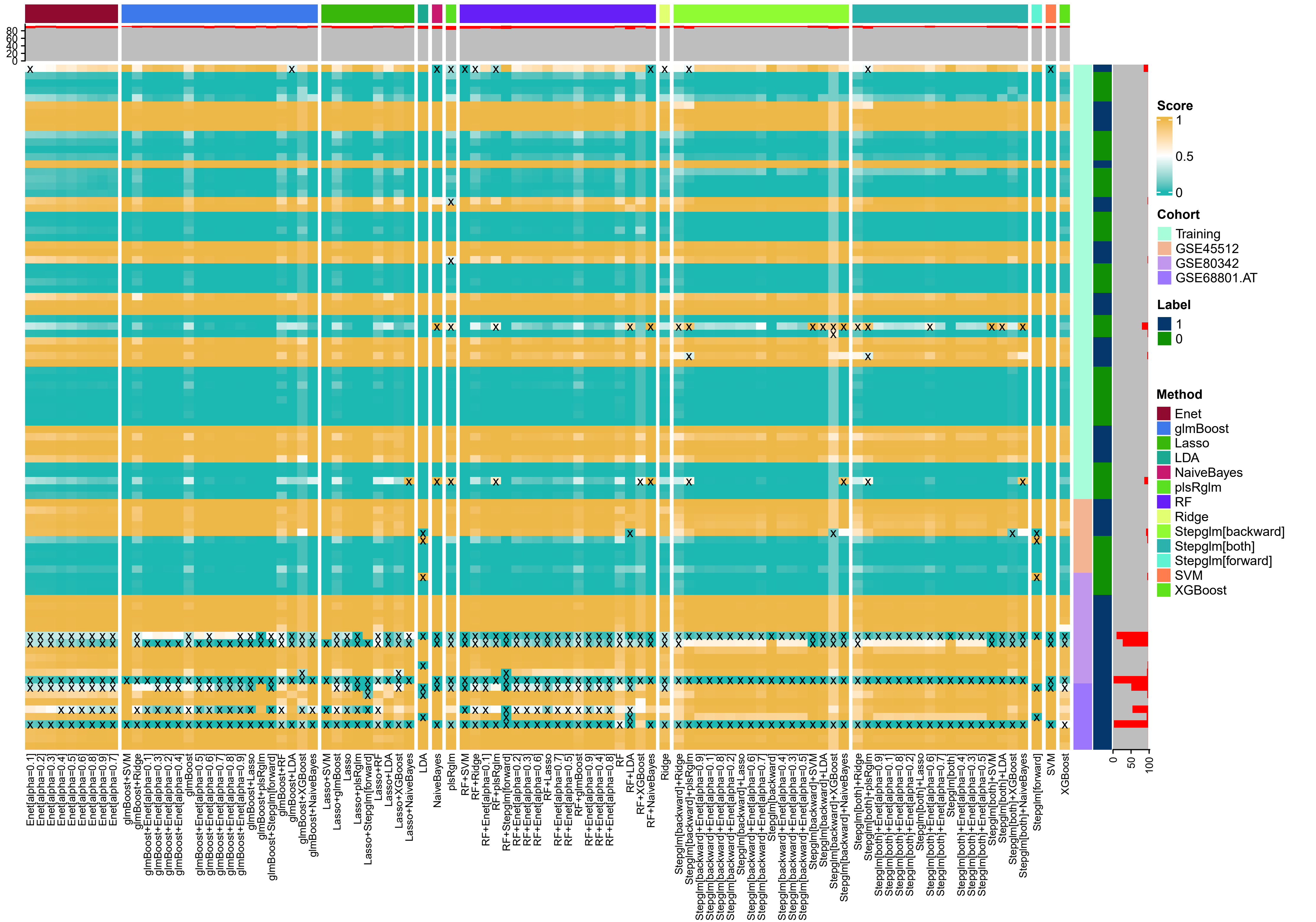

Score
1
0.5
0
Cohort
Training
GSE45512
GSE80342
GSE68801.AT
Label
1
0
Method
Enet
glmBoost
Lasso
LDA
NaiveBayes
plsRglm
RF
Ridge
Stepglm[backward]
Stepglm[both]
Stepglm[forward]
SVM
XGBoost
80
60
40
20
0
0
50
100
Enet[alpha=0.1]
Enet[alpha=0.2]
Enet[alpha=0.3]
Enet[alpha=0.4]
Enet[alpha=0.5]
Enet[alpha=0.6]
Enet[alpha=0.8]
Enet[alpha=0.9]
Enet[alpha=0.7]
glmBoost+SVM
glmBoost+Ridge
glmBoost+Enet[alpha=0.1]
glmBoost+Enet[alpha=0.3]
glmBoost+Enet[alpha=0.2]
glmBoost+Enet[alpha=0.4]
glmBoost
glmBoost+Enet[alpha=0.5]
glmBoost+Enet[alpha=0.6]
glmBoost+Enet[alpha=0.7]
glmBoost+Enet[alpha=0.8]
glmBoost+Enet[alpha=0.9]
glmBoost+Lasso
glmBoost+plsRglm
glmBoost+Stepglm[forward]
glmBoost+RF
glmBoost+LDA
glmBoost+XGBoost
glmBoost+NaiveBayes
Lasso+SVM
Lasso+glmBoost
Lasso
Lasso+plsRglm
Lasso+Stepglm[forward]
Lasso+RF
Lasso+LDA
Lasso+XGBoost
Lasso+NaiveBayes
LDA
NaiveBayes
plsRglm
RF+SVM
RF+Ridge
RF+Enet[alpha=0.1]
RF+plsRglm
RF+Stepglm[forward]
RF+Enet[alpha=0.2]
RF+Enet[alpha=0.3]
RF+Enet[alpha=0.6]
RF+Lasso
RF+Enet[alpha=0.7]
RF+Enet[alpha=0.5]
RF+glmBoost
RF+Enet[alpha=0.9]
RF+Enet[alpha=0.4]
RF+Enet[alpha=0.8]
RF
RF+LDA
RF+XGBoost
RF+NaiveBayes
Ridge
Stepglm[backward]+Ridge
Stepglm[backward]+plsRglm
Stepglm[backward]+Enet[alpha=0.9]
Stepglm[backward]+Enet[alpha=0.1]
Stepglm[backward]+Enet[alpha=0.8]
Stepglm[backward]+Enet[alpha=0.2]
Stepglm[backward]+Lasso
Stepglm[backward]+Enet[alpha=0.6]
Stepglm[backward]+Enet[alpha=0.7]
Stepglm[backward]
Stepglm[backward]+Enet[alpha=0.4]
Stepglm[backward]+Enet[alpha=0.3]
Stepglm[backward]+Enet[alpha=0.5]
Stepglm[backward]+SVM
Stepglm[backward]+LDA
Stepglm[backward]+XGBoost
Stepglm[backward]+NaiveBayes
Stepglm[both]+Ridge
Stepglm[both]+plsRglm
Stepglm[both]+Enet[alpha=0.9]
Stepglm[both]+Enet[alpha=0.1]
Stepglm[both]+Enet[alpha=0.8]
Stepglm[both]+Enet[alpha=0.2]
Stepglm[both]+Lasso
Stepglm[both]+Enet[alpha=0.6]
Stepglm[both]+Enet[alpha=0.7]
Stepglm[both]
Stepglm[both]+Enet[alpha=0.4]
Stepglm[both]+Enet[alpha=0.3]
Stepglm[both]+Enet[alpha=0.5]
Stepglm[both]+SVM
Stepglm[both]+LDA
Stepglm[both]+XGBoost
Stepglm[both]+NaiveBayes
Stepglm[forward]
SVM
XGBoost

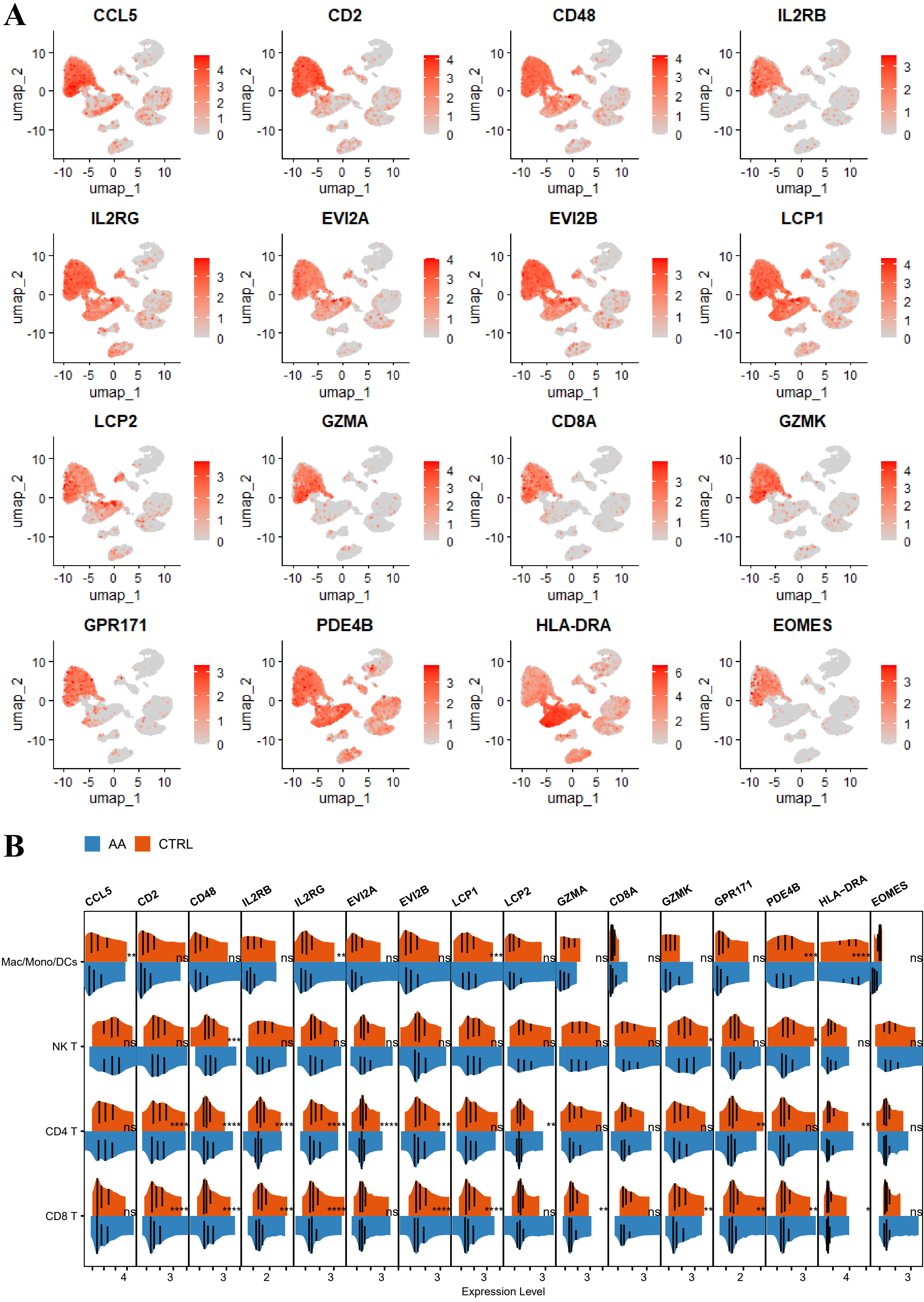
A
CCL5
CD2
CD48
IL2RB
IL2RG
EVI2A
EVI2B
LCP1
LCP2
GZMA
CD8A
GZMK
GPR171
PDE4B
HLA-DRA
EOMES
umap_1
umap_2
B
AA
CTRL
Mac/Mono/DCs
NK T
CD4 T
CD8 T
Expression Level

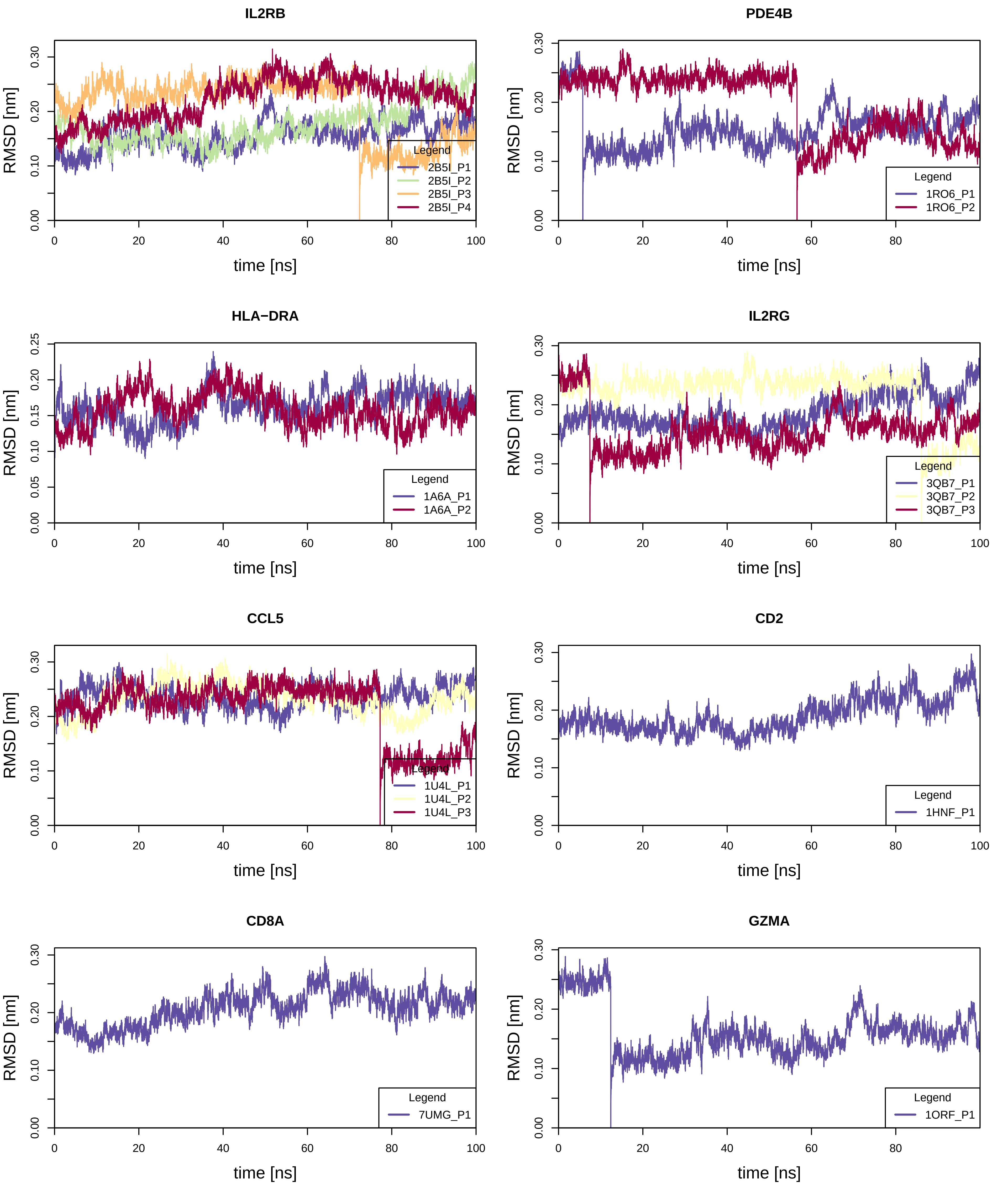
IL2RB
RMSD [nm]
time [ns]
Legend
2B5I_P1
2B5I_P2
2B5I_P3
2B5I_P4
PDE4B
RMSD [nm]
time [ns]
Legend
1RO6_P1
1RO6_P2
HLA−DRA
RMSD [nm]
time [ns]
Legend
1A6A_P1
1A6A_P2
IL2RG
RMSD [nm]
time [ns]
Legend
3QB7_P1
3QB7_P2
3QB7_P3
CCL5
RMSD [nm]
time [ns]
Legend
1U4L_P1
1U4L_P2
1U4L_P3
CD2
RMSD [nm]
time [ns]
Legend
1HNF_P1
CD8A
RMSD [nm]
time [ns]
Legend
7UMG_P1
GZMA
RMSD [nm]
time [ns]
Legend
1ORF_P1

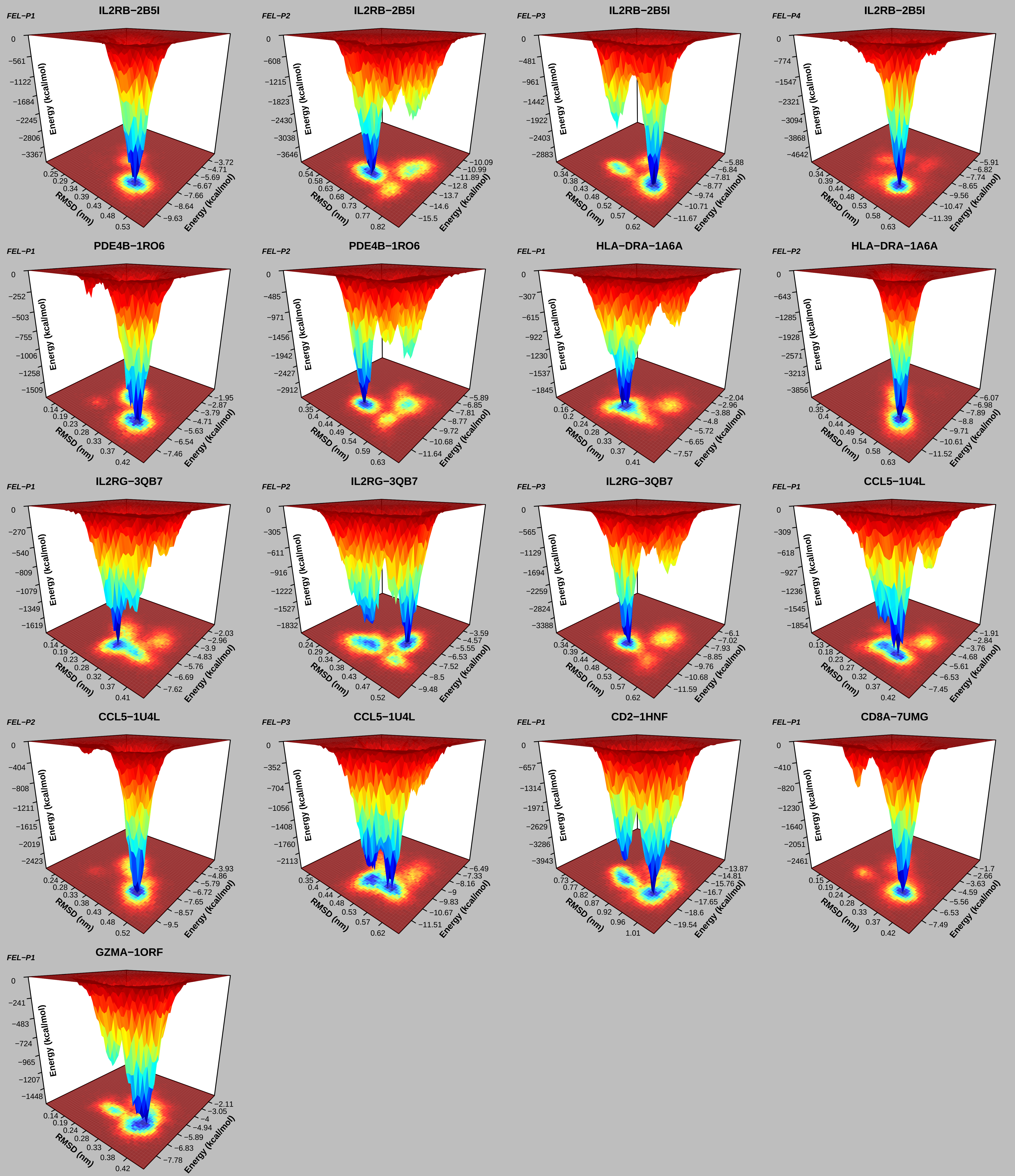
FEL−P1
IL2RB−2B5I
0 −561 −1122 −1684 −2245 −2806 −3367
Energy (kcal/mol)
0.25 0.29 0.34 0.39 0.43 0.48 0.53
RMSD (nm)
−3.72 −4.71 −5.69 −6.67 −7.66 −8.64 −9.63
Energy (kcal/mol)
FEL−P2
IL2RB−2B5I
0 −608 −1215 −1823 −2430 −3038 −3646
Energy (kcal/mol)
0.54 0.58 0.63 0.68 0.73 0.77 0.82
RMSD (nm)
−10.09 −10.99 −11.89 −12.8 −13.7 −14.6 −15.5
Energy (kcal/mol)
FEL−P3
IL2RB−2B5I
0 −481 −961 −1442 −1922 −2403 −2883
Energy (kcal/mol)
0.34 0.38 0.43 0.48 0.52 0.57 0.62
RMSD (nm)
−5.88 −6.84 −7.81 −8.77 −9.74 −10.71 −11.67
Energy (kcal/mol)
FEL−P4
IL2RB−2B5I
0 −774 −1547 −2321 −3094 −3868 −4642
Energy (kcal/mol)
0.34 0.39 0.44 0.48 0.53 0.58 0.63
RMSD (nm)
−5.91 −6.82 −7.74 −8.65 −9.56 −10.47 −11.39
Energy (kcal/mol)
FEL−P1
PDE4B−1RO6
0 −252 −503 −755 −1006 −1258 −1509
Energy (kcal/mol)
0.14 0.19 0.23 0.28 0.33 0.37 0.42
RMSD (nm)
−1.95 −2.87 −3.79 −4.71 −5.63 −6.54 −7.46
Energy (kcal/mol)
FEL−P2
PDE4B−1RO6
0 −485 −971 −1456 −1942 −2427 −2912
Energy (kcal/mol)
0.35 0.4 0.44 0.49 0.54 0.59 0.63
RMSD (nm)
−5.89 −6.85 −7.81 −8.77 −9.72 −10.68 −11.64
Energy (kcal/mol)
FEL−P1
HLA−DRA−1A6A
0 −307 −615 −922 −1230 −1537 −1845
Energy (kcal/mol)
0.16 0.2 0.24 0.28 0.33 0.37 0.41
RMSD (nm)
−2.04 −2.96 −3.88 −4.8 −5.72 −6.65 −7.57
Energy (kcal/mol)
FEL−P2
HLA−DRA−1A6A
0 −643 −1285 −1928 −2571 −3213 −3856
Energy (kcal/mol)
0.35 0.4 0.44 0.49 0.54 0.58 0.63
RMSD (nm)
−6.07 −6.98 −7.89 −8.8 −9.71 −10.61 −11.52
Energy (kcal/mol)
FEL−P1
IL2RG−3QB7
0 −270 −540 −809 −1079 −1349 −1619
Energy (kcal/mol)
0.14 0.19 0.23 0.28 0.32 0.37 0.41
RMSD (nm)
−2.03 −2.96 −3.9 −4.83 −5.76 −6.69 −7.62
Energy (kcal/mol)
FEL−P2
IL2RG−3QB7
0 −305 −611 −916 −1222 −1527 −1832
Energy (kcal/mol)
0.24 0.29 0.34 0.38 0.43 0.47 0.52
RMSD (nm)
−3.59 −4.57 −5.55 −6.53 −7.52 −8.5 −9.48
Energy (kcal/mol)
FEL−P3
IL2RG−3QB7
0 −565 −1129 −1694 −2259 −2824 −3388
Energy (kcal/mol)
0.34 0.39 0.44 0.48 0.53 0.57 0.62
RMSD (nm)
−6.1 −7.02 −7.93 −8.85 −9.76 −10.68 −11.59
Energy (kcal/mol)
FEL−P1
CCL5−1U4L
0 −309 −618 −927 −1236 −1545 −1854
Energy (kcal/mol)
0.13 0.18 0.23 0.27 0.32 0.37 0.42
RMSD (nm)
−1.91 −2.84 −3.76 −4.68 −5.61 −6.53 −7.45
Energy (kcal/mol)
FEL−P2
CCL5−1U4L
0 −404 −808 −1211 −1615 −2019 −2423
Energy (kcal/mol)
0.24 0.28 0.33 0.38 0.43 0.48 0.52
RMSD (nm)
−3.93 −4.86 −5.79 −6.72 −7.65 −8.57 −9.5
Energy (kcal/mol)
FEL−P3
CCL5−1U4L
0 −352 −704 −1056 −1408 −1760 −2113
Energy (kcal/mol)
0.35 0.4 0.44 0.48 0.53 0.57 0.62
RMSD (nm)
−6.49 −7.33 −8.16 −9 −9.83 −10.67 −11.51
Energy (kcal/mol)
FEL−P1
CD2−1HNF
0 −657 −1314 −1971 −2629 −3286 −3943
Energy (kcal/mol)
0.73 0.77 0.82 0.87 0.92 0.96 1.01
RMSD (nm)
−13.87 −14.81 −15.76 −16.7 −17.65 −18.6 −19.54
Energy (kcal/mol)
FEL−P1
CD8A−7UMG
0 −410 −820 −1230 −1640 −2051 −2461
Energy (kcal/mol)
0.15 0.19 0.24 0.28 0.33 0.37 0.42
RMSD (nm)
−1.7 −2.66 −3.63 −4.59 −5.56 −6.53 −7.49
Energy (kcal/mol)
FEL−P1
GZMA−1ORF
0 −241 −483 −724 −965 −1207 −1448
Energy (kcal/mol)
0.14 0.19 0.24 0.28 0.33 0.38 0.42
RMSD (nm)
−2.11 −3.05 −4 −4.94 −5.89 −6.83 −7.78
Energy (kcal/mol)

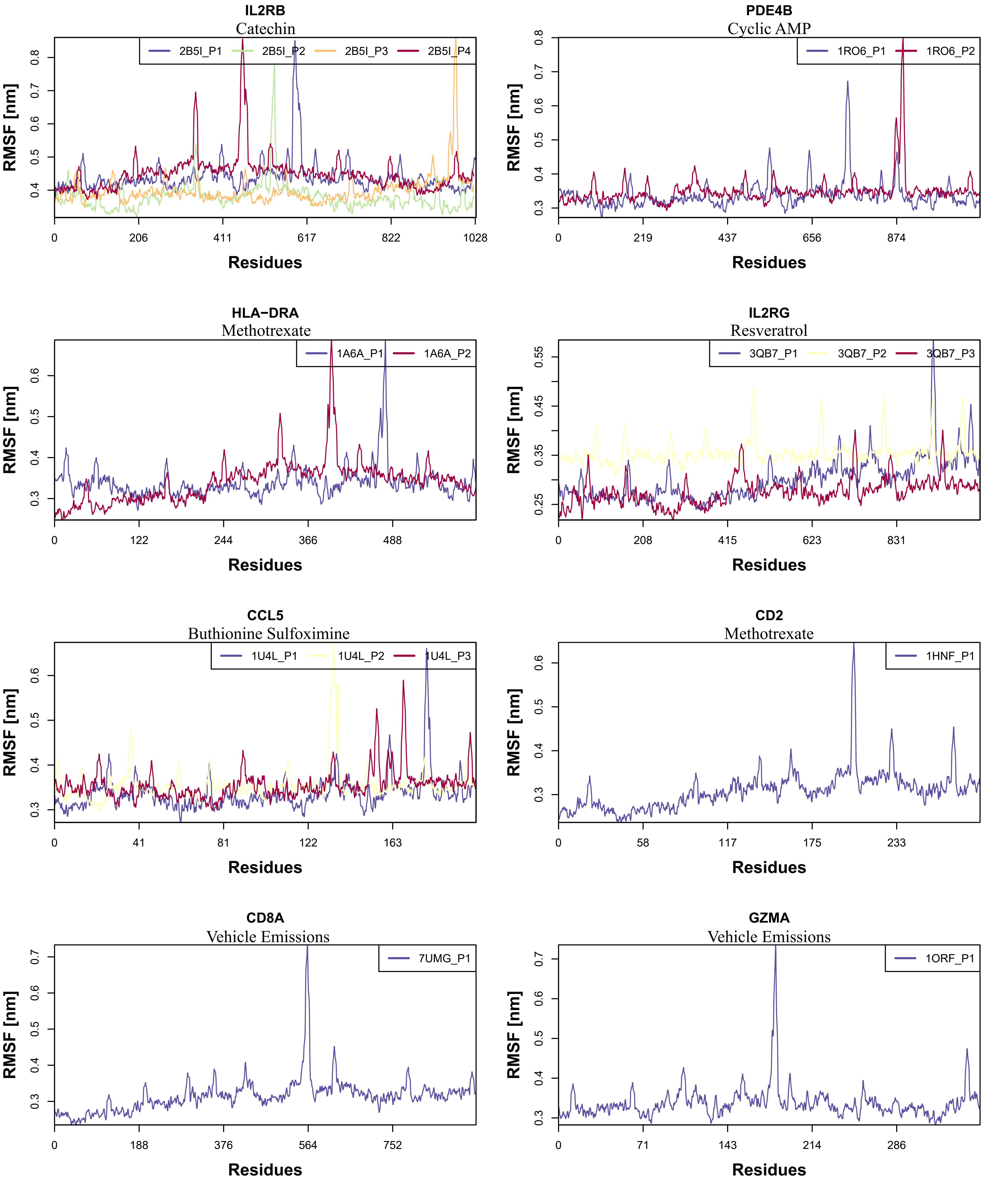
IL2RB
Catechin
2B5I_P1
2B5I_P2
2B5I_P3
2B5I_P4
RMSF [nm]
0.4
0.5
0.6
0.7
0.8
0
206
411
617
822
1028
Residues
PDE4B
Cyclic AMP
1RO6_P1
1RO6_P2
RMSF [nm]
0.3
0.4
0.5
0.6
0.7
0.8
0
219
437
656
874
Residues
HLA−DRA
Methotrexate
1A6A_P1
1A6A_P2
RMSF [nm]
0.3
0.4
0.5
0.6
0
122
244
366
488
Residues
IL2RG
Resveratrol
3QB7_P1
3QB7_P2
3QB7_P3
RMSF [nm]
0.25
0.35
0.45
0.55
0
208
415
623
831
Residues
CCL5
Buthionine Sulfoximine
1U4L_P1
1U4L_P2
1U4L_P3
RMSF [nm]
0.3
0.4
0.5
0.6
0
41
81
122
163
Residues
CD2
Methotrexate
1HNF_P1
RMSF [nm]
0.3
0.4
0.5
0.6
0
58
117
175
233
Residues
CD8A
Vehicle Emissions
7UMG_P1
RMSF [nm]
0.3
0.4
0.5
0.6
0.7
0
188
376
564
752
Residues
GZMA
Vehicle Emissions
1ORF_P1
RMSF [nm]
0.3
0.4
0.5
0.6
0.7
0
71
143
214
286
Residues

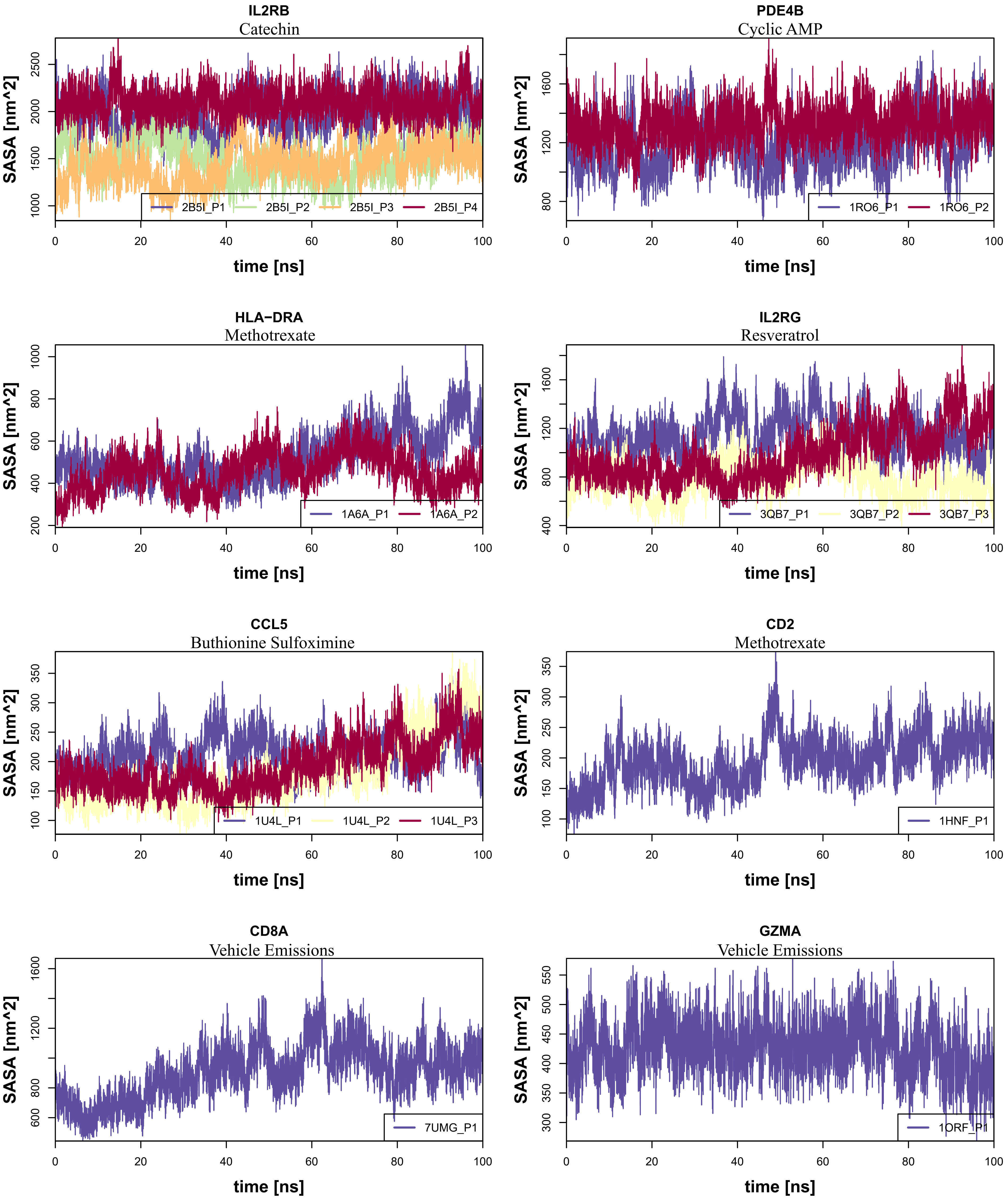
IL2RB
Catechin
SASA [nm^2]
time [ns]
2B5I_P1
2B5I_P2
2B5I_P3
2B5I_P4
PDE4B
Cyclic AMP
SASA [nm^2]
time [ns]
1RO6_P1
1RO6_P2
HLA−DRA
Methotrexate
SASA [nm^2]
time [ns]
1A6A_P1
1A6A_P2
IL2RG
Resveratrol
SASA [nm^2]
time [ns]
3QB7_P1
3QB7_P2
3QB7_P3
CCL5
Buthionine Sulfoximine
SASA [nm^2]
time [ns]
1U4L_P1
1U4L_P2
1U4L_P3
CD2
Methotrexate
SASA [nm^2]
time [ns]
1HNF_P1
CD8A
Vehicle Emissions
SASA [nm^2]
time [ns]
7UMG_P1
GZMA
Vehicle Emissions
SASA [nm^2]
time [ns]
1ORF_P1
